# High-Altitude Andean H194R *HIF2A* Allele Is a Hypomorphic Allele

**DOI:** 10.1093/molbev/msad162

**Published:** 2023-07-18

**Authors:** Kelsey Jorgensen, Daisheng Song, Julien Weinstein, Obed A Garcia, Laurel N Pearson, María Inclán, Maria Rivera-Chira, Fabiola León-Velarde, Melisa Kiyamu, Tom D Brutsaert, Abigail W Bigham, Frank S Lee

**Affiliations:** Department of Anthropology, University of California, Los Angeles, CA, USA; Department of Pathology and Laboratory Medicine, University of Pennsylvania Perelman School of Medicine, Philadelphia, PA, USA; Department of Anthropology, The University of Michigan, Ann Arbor, MI, USA; Department of Biomedical Data Science, Stanford University, Stanford, CA, USA; Department of Anthropology, The Pennsylvania State University, State College, PA, USA; División de. Estudios Políticos, Centro de Investigación y Docencia Económicas, Mexico City, CDMX, Mexico; Departamento de Ciencias Biológicas y Fisiológicas, Laboratorios de Investigación y Desarrollo, Facultad de Ciencias y Filosofía, Universidad Peruana Cayetano Heredia, Lima, Lima, Peru; Departamento de Ciencias Biológicas y Fisiológicas, Laboratorios de Investigación y Desarrollo, Facultad de Ciencias y Filosofía, Universidad Peruana Cayetano Heredia, Lima, Lima, Peru; Departamento de Ciencias Biológicas y Fisiológicas, Laboratorios de Investigación y Desarrollo, Facultad de Ciencias y Filosofía, Universidad Peruana Cayetano Heredia, Lima, Lima, Peru; Department of Exercise Science, Syracuse University, Syracuse, NY, USA; Department of Anthropology, University of California, Los Angeles, CA, USA; Department of Pathology and Laboratory Medicine, University of Pennsylvania Perelman School of Medicine, Philadelphia, PA, USA

**Keywords:** Andean evolution, *EPAS1*, *HIF2A*, high-altitude adaptation, hypoxia, hypoxia-inducible factor

## Abstract

For over 10,000 years, Andeans have resided at high altitude where the partial pressure of oxygen challenges human survival. Recent studies have provided evidence for positive selection acting in Andeans on the *HIF2A* (also known as *EPAS1*) locus, which encodes for a central transcription factor of the hypoxia-inducible factor pathway. However, the precise mechanism by which this allele might lead to altitude-adaptive phenotypes, if any, is unknown. By analyzing whole genome sequencing data from 46 high-coverage Peruvian Andean genomes, we confirm evidence for positive selection acting on *HIF2A* and a unique pattern of variation surrounding the Andean-specific single nucleotide variant (SNV), rs570553380, which encodes for an H194R amino acid substitution in HIF-2α. Genotyping the Andean-associated SNV rs570553380 in a group of 299 Peruvian Andeans from Cerro de Pasco, Peru (4,338 m), reveals a positive association with increased fraction of exhaled nitric oxide, a marker of nitric oxide biosynthesis. In vitro assays show that the H194R mutation impairs binding of HIF-2α to its heterodimeric partner, aryl hydrocarbon receptor nuclear translocator. A knockin mouse model bearing the H194R mutation in the *Hif2a* gene displays decreased levels of hypoxia-induced pulmonary *Endothelin-1* transcripts and protection against hypoxia-induced pulmonary hypertension. We conclude the Andean H194R *HIF2A* allele is a hypomorphic (partial loss of function) allele.

## Introduction

Human populations have lived for thousands of years at high altitude, a key feature of which is hypobaric low oxygen or hypoxia (Beall 2014; Moore 2017). The challenges of living under chronic hypoxia include optimizing delivery of oxygen to tissues of the body, adjusting demand for oxygen by these tissues, and avoiding unintended consequences arising from pathophysiologic responses at high altitude that would otherwise be normal at low altitude (Storz et al. 2010). Long-term resident high-altitude human populations including Andeans and Tibetans have overcome these challenges through unique physiological adaptations, raising the question of whether they have employed different or similar evolutionary strategies to survive at high altitude (Beall 2007). Recent work has indicated that genetic changes play a role in this adaptation. In both populations, genetic signatures have been identified in genes of the hypoxia-inducible factor (HIF) pathway, the central mammalian pathway that mediates the transcriptional response to hypoxia (Bigham et al. 2010; Bigham and Lee 2014).

HIF is a heterodimeric transcription factor that consists of an α subunit, of which HIF-1α and HIF-2α are the main paralogs, and a common β subunit (also known as aryl hydrocarbon receptor nuclear translocator or ARNT) (Kaelin and Ratcliffe 2008; Majmundar et al. 2010; Semenza 2014; Schödel and Ratcliffe 2019). Under normoxic conditions, the enzyme prolyl hydroxylase domain protein 2 (PHD2) site-specifically prolyl hydroxylates HIF-α, allowing for interaction with the von Hippel–Lindau (VHL) protein, which, in turn, targets HIF-α for degradation by the ubiquitin–proteasome pathway (Ivan et al. 2001; Jaakkola et al. 2001; Yu et al. 2001). Under hypoxia, this posttranslational modification is arrested, leading to stabilization of HIF-α, its dimerization with ARNT, and the activation of HIF target genes. HIF-1α and HIF-2α have overlapping as well as distinctive targets (Keith et al. 2012). For example, HIF-1α targets include genes that encode for enzymes of glycolysis (Semenza 1999). HIF-2α gene targets include *EPO*, which encodes the central hormone regulator of red cell mass, and *EDN1*, which encodes the potent vasoconstrictor Endothelin-1 (Scortegagna et al. 2005; Hickey et al. 2010). HIF-2α also plays a role in nitric oxide (NO) metabolism, ventilatory control, and angiogenesis (Keith et al. 2012; Hodson et al. 2016).

NO is a potent vasodilator that contributes to pulmonary artery pressure (PAP) regulation and is known to have beneficial effects in the management of pulmonary hypertension (Hambly et al. 2016; Lau et al. 2017). Increased levels of systemic NO may be an adaptive response to high-altitude hypoxia to promote blood flow and therefore increase tissue oxygen delivery (Beall et al. 2001; Hoit et al. 2005; Erzurum et al. 2007). In the lung, the fractional concentration of exhaled NO (FeNO) is a noninvasive clinical measurement of airway inflammation. Elevated levels of FeNO may indicate increased levels of the vasodilator NO in the lungs and therefore may be indicative of HIF-2α regulation of NO metabolism.

The high-altitude Tibetan population shows evidence of selection on two genes of the HIF pathway, *PHD2* (also known as *EGLN1*) and *HIF2A* (also known as *EPAS1*) (Bigham and Lee 2014). The Tibetan *PHD2* allele shows partial loss of function (hypomorphic) properties (Song et al. 2014, 2020). The Tibetan *HIF2A* allele is correlated with lower hemoglobin (Hb) levels (Beall et al. 2010; Yi et al. 2010). Hb levels are a reflection of red cell mass, which is controlled by HIF-2α regulated EPO production (Lee and Percy 2011; Lappin and Lee 2019). Moreover, the Tibetan *HIF2A* allele is correlated with lower right ventricular pressures (Peng et al. 2017). *HIF2A* partial gain-of-function (hypermorphic) mutations are associated with erythrocytosis and pulmonary hypertension in humans and in mice (Gale et al. 2008; Percy et al. 2008; Tan et al. 2013). Taken together, the aforementioned observations on the Tibetan *HIF2A* allele are consistent with it being a hypomorphic allele. In contrast to the Tibetan *PHD2* allele, which is characterized by two coding sequence (CDS) mutations that flank a zinc finger motif in the PHD2 protein (Xiang et al. 2013; Lorenzo et al. 2014), the Tibetan *HIF2A* allele does not contain any CDS mutations. Select Tibetan *HIF2A* single nucleotide variants (SNVs), in reporter gene assays, lead to decreased transcriptional activity (Gray et al. 2022). It remains a challenging task to identify which combination of Tibetan *HIF2A* SNVs is responsible for in vivo phenotypic changes in this population.

Like Tibetans, Andeans display evidence for selection on both *PHD2* (Bigham et al. 2010; Foll et al. 2014) and *HIF2A* (Foll et al. 2014; Eichstaedt et al. 2017). The Andean *PHD2* allele is associated with higher VO_2_max, a measure of aerobic capacity (Brutsaert et al. 2019) whereas, to date, the Andean *HIF2A* allele has not been associated with an altitude-adaptive phenotype in this population. In contrast to the Tibetan *HIF2A* allele, which lacks any CDS changes, the Andean *HIF2A* allele is characterized by a nucleotide change (rs570553380) that produces an H194R missense mutation in the HIF-2α protein (Eichstaedt et al. 2017). The frequency of this mutation increases from 0.06 in Indigenous Argentine lowlanders to 0.32 in Andean Collas from Argentina (Eichstaedt et al. 2017). Among Peruvians residing in Lima (PEL) (altitude of 500 ft = 150 m) from the 1000 Genomes Project (1KG) who possess some degree of Andean ancestry, the G missense change is 7%. This mutation is not present in other 1KG populations.

These observations make it of substantial interest to determine if the Andean H194R mutation is associated with the fraction of exhaled NO (FeNO), a marker of NO biosynthesis, and/or Hb concentration, if it produces any functional change in the HIF-2α protein, and if so, whether it is hypermorphic or hypomorphic.

## Results

### Evidence for Peruvian Andean *HIF2A* Positive Selection

We recruited 301 high-altitude Peruvian Andeans between the ages of 18 and 35 years from the city of Cerro de Pasco, Peru (4,338 m) (Brutsaert et al. 2019; Childebayeva et al. 2019). All study participants were healthy, nonsmoking male (*n* = 156) and female (*n* = 145) individuals who self-identified as Quechua. It is important to note that Quechua is a language designation reflecting Indigenous Andean ancestry, but not necessarily Quechua ancestry per se. Today, distinct Andean ethnic groups have been subsumed under the Quechua census label (Sandoval et al. 2016). Collectively, they are the descendants of Indigenous Andeans who have resided on the Altiplano for upwards of 11,000 years (Rademaker et al. 2014). We selected 48 individuals from the full cohort of 301 high-altitude Peruvian Andeans for whole genome sequencing (WGS) performed on an Illumina Nova Seq using an S4 flow cell at the University of Michigan's DNA sequencing core. In addition, we obtained WGS data from 48 Maya language speakers (Tzeltal, Tzotzil, and Ch’ol) recruited from Palenque, Chiapas, Mexico (13 m). All study participants provided informed consent for the study of high-altitude adaptation. Andean and Maya individuals selected for WGS displayed low evidence of European admixture based on principal component analysis (PCA) (Purcell et al. 2007) and fastStructure analysis (Raj et al. 2014) of Affymetrix (Santa Clara, CA) Axiom Biobank array data (Brutsaert et al. 2019) (supplementary fig. S1, Supplementary Material online) and were unrelated at the first, second, or third degree (Manichaikul et al. 2010). Two Peruvian Andean samples with data missingness >10% were removed from downstream analysis. All 48 Maya language speakers passed initial quality control filtering.

We identified 284 SNVs in *HIF2A* of which 181 were known and 103 were novel (supplementary table S1 and fig. S2*A*, Supplementary Material online). Of these total SNVs, 272 were intronic and 4 were in the CDS, including the H194R A to G missense mutation located in exon 6 that affects a residue in a conserved, structured region of the HIF-2α protein. The H194R (rs570553380) G allele frequency was 5.6% among Peruvian Andeans and not present among Mexican Maya language speakers. The three additional CDS variants were known SNVs, two of which were missense mutations affecting residues predicted to reside in unstructured regions of the HIF-2α protein (rs59901247 [T766P] and rs61518065 [P785T], Peruvian Andean minor allele frequencies [MAFs] = 0.01) and one of which was a synonymous change (rs184760160, Peruvian Andean MAF = 0.01). The 5ʹ and 3ʹ untranslated regions contained one and seven known SNVs, respectively, all with relatively low Peruvian Andean MAFs.

We calculated statistical evidence of recent positive selection in *HIF2A* and 50 kb upstream and downstream of the CDS coordinates using the population branch statistic (PBS), the cross-population number of segregating sites by length (XP-nSL), and a composite test of selection (Eichstaedt et al. 2017). We identified 32 significant SNVs for PBS ranging from PBS = 0.07 to PBS = 0.14 (supplementary table S2 and fig. S2*B*, Supplementary Material online). Of these, five were within *HIF2A*: two novel intronic variants currently without rsIDS, (hg38 2:46,326,827) and (hg38 2:46,328,082), and three known intronic variants, rs182554252, rs187003095, and rs4952821, the latter of which is located in a potential *HIF2A* enhancer (www.ensembl.org). For XP-nSL, we identified four significant SNVs that fell within *HIF2A* using a 1% significance threshold of XP-nSL = 2.53 based on a genome-wide empirical distribution (supplementary table S3 and fig. S2*C*, Supplementary Material online). Each significant XP-nSL SNV was located adjacent (<12 kb) to the missense mutation of interest, H194R (rs570553380) at hg38 position 46360892 (hg19 46588031). Using the composite method of selection, we identified 14 significant SNVs upstream, downstream, and within the CDS (supplementary table S4, Supplementary Material online). H194R did not emerge as a statistically significant variant for the three tests considered.

**Fig. 1.**
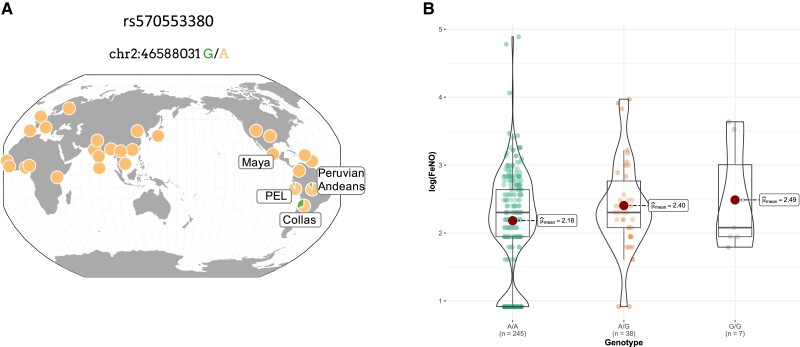
(*A*) Global allele frequency of the H194R missense variant depicted for Peruvian Andeans, Mexican Maya, Argentine Collas, and 1000 Genomes Project populations. The ancestral allele (A) and the derived allele (G) are shown. This image was downloaded from the Geography of Genetic Variants Browser (Marcus and Novembre 2017). (*B*) Violin plots of the natural log of the fractional exhaled NO (FeNO) by H194R genotype. Individual data points for each genotype are shown as filled circles. Mean natural log FeNO values are shown as a single solid central circle for each genotype with the mean values presented for each. H194R genotype was significantly associated with FeNO using an additive model (*β* = 0.20 [95% CI 0.02–0.38], *P* = 0.02, *R*^2^ = 0.13) and a dominant model (*β* = 0.26 [95% CI 0.04–0.48], *P* = 0.02, *R*^2^ = 0.14). Models controlled for sex and population structure.

It remains striking that the H194R G variant is a private allele only identified to date in the Andean region. No other global population possesses this derived missense variant (fig. 1*A*). To understand if other genetic variants follow this same pattern, we identified all unique nonsynonymous variants with a MAF ≥ 5% in Peruvian Andeans. In total, there were 5,702,635 Peruvian Andean SNVs, of which 7,621 were nonsynonymous variants. Of these, 21 were nonsynonymous variants with a MAF ≥ 5%, found only among Peruvian Andeans and absent from 1KG populations (aside from PEL) and Mexican Maya (supplementary table S5, Supplementary Material online). The H194R variant was the singular nonsynonymous SNV (MAF ≥ 5%) near *HIF2A*. In conjunction with the previously reported early-stage selection among high-altitude Argentine Collas (Eichstaedt et al. 2017), we hypothesize that *HIF2A* has been the target of past natural selection, and that the H194R-derived G allele is a new mutation, unique to the Andean region, that may contribute to altitude-adaptive phenotypes.

### Andean *HIF2A* Is Associated With FeNO

We characterized the H194R missense SNV (rs570553380) in our full cohort of 301 Peruvian Andeans using polymerase chain reaction (PCR) coupled with diagnostic restriction enzyme digestion. We obtained genotype data for 300 individuals. A/G heterozygotes were confirmed with Sanger sequencing. The H194R G frequency was 9%, slightly greater than the frequency of this allele among 1KG PEL (7%) and the frequency in our WGS subsample (5.6%). We tested for an association between H194R genotype and two phenotypes: Hb concentration and FeNO. Hb concentration was available for 299 of the 301 study participants. Average Hb concentration was 17.70 g/dl ± 2.18 (SD), with 19.16 ± 1.70 for males and 16.14 ± 1.43 for females. For FeNO, we obtained data for 300 of the 301 study participants. Average FeNO was 12.03 ppb ± 12.21 for all participants, 14.16 ± 12.55 and 9.75 ± 11.46 for males and females, respectively (table 1). Genotype–phenotype association testing was performed using data from 290 individuals for FeNO and 289 individuals for Hb. These sample sizes reflect the total number of individuals for each phenotype who had associated genotype data and were unrelated at the second degree or higher level. FeNO measures did not follow a normal distribution and were log transformed prior to association testing. H194R was significantly associated with FeNO using a linear regression model controlling for sex and population structure using the first five PCs estimated from Affymetrix Biobank Array data (additive model *β* = 0.20 [95% confidence interval {CI} 0.02–0.38], *P* = 0.03, *R*^2^ = 0.13; dominant model *β* = 0.26 [95% CI 0.04–0.48], *P* = 0.02, *R*^2^ = 0.14, *n* = 290) (fig. 1*B*). Average log-transformed FeNO was 2.18 ppb (range = 0.92–4.89) for AA genotypes, 2.40 ppb (range = 0.92–3.97) for AG genotypes, and 2.49 ppb (range = 1.79–3.63) for GG genotypes. We did not detect a significant association between H194R genotype and Hb concentration in models that controlled for sex and population substructure.

**Table 1.** Participant Characteristics.

	All (301)	Male (156)	Female (145)
Age, years*	24.29 ± 4.99	23.79 ± 4.82	24.83 ± 5.13
Ht, cm*	158.17 ± 8.14	164.02 ± 5.61	151.87 ± 5.21
Wt, kg*	59.49 ± 8.2	62.35 ± 7.75	56.43 ± 7.57
Sex	0.48 [0.43–0.54]	NA	NA
Hb, g/dl*	17.70 ± 2.18	19.16 ± 1.70	16.14 ± 1.43
FeNO, ppb*	12.03 ± 12.21	14.16 ± 12.55	9.75 ± 11.46

Note.—Data are means ± SD, 95% CI for proportions in brackets, and sample sizes shown in parenthesis. NA, not applicable.

* *P* ≤ 0.05.

### Andean HIF-2α Produces a Partial Loss of Function In Vitro

**Fig. 2.**
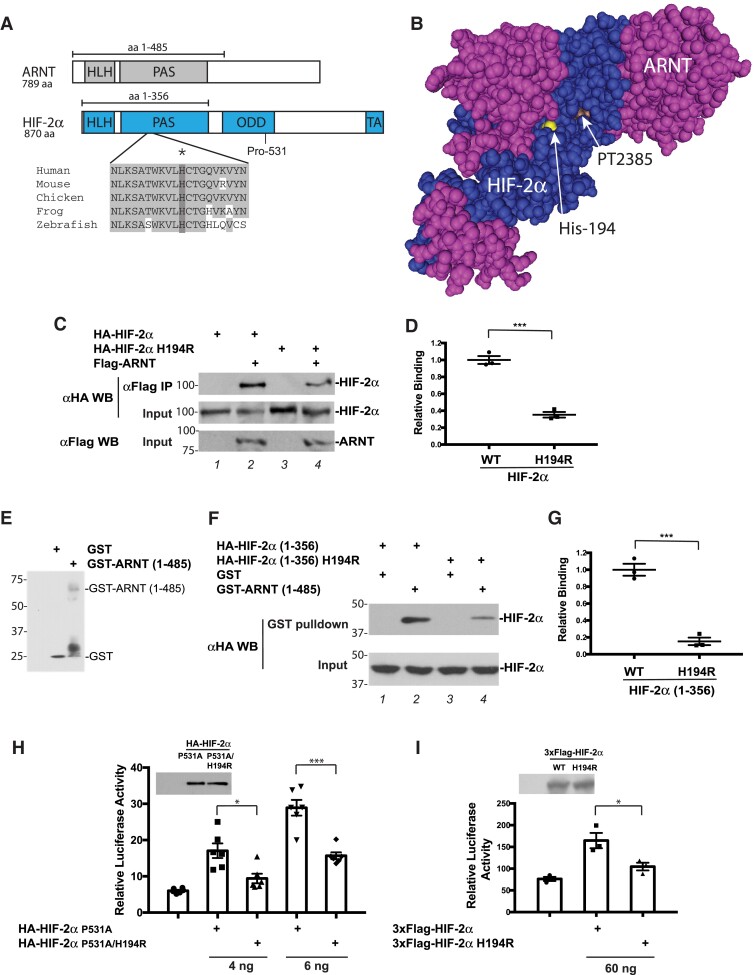
H194R HIF-2α displays impaired heterodimerization with ARNT and decreased transcriptional activity. (*A*) Top, diagram of ARNT and HIF-2α. Primary site of regulatory prolyl hydroxylation in HIF-2α, Pro-531, is shown. Bottom, comparison of HIF-2α amino acids 184–203 (human numbering) across species. Shading indicates conserved residues. His-194 is indicated by an asterisk and dark shading. (*B*) Structure of the HIF-2α:ARNT heterodimer (HLH and PAS domains) bound to PT2385 (Wu et al. 2019). HIF-2α residue His-194, near the interface, is highlighted as indicated. PT2385, which binds to a pocket in HIF-2α, is indicated. (*C*) Coimmunoprecipitation studies of in vitro translated WT or H194R hemagglutinin (HA)-tagged HIF-2α with in vitro translated Flag-ARNT. Positions of molecular weight markers are shown to the left. (*D*) Quantitation of coimmunoprecipitation experiments shown in (*C*). *n* = 3 per group; ***P < 0.001. (*E*) Anti-GST western blot of GST and GST-ARNT (1–585). Positions of these proteins are shown to the right. Positions of molecular weight markers are shown to the left. (*F*) GST pull-down assays of WT or H194R HA-HIF-2α (1–356) from transfected HEK293FT cells. Positions of molecular weight markers are shown to the left. (*G*) Quantitation of GST pull-down experiments shown in (*F*). *n* = 3 per group; ***P < 0.001. (*H* and *I*) HRE reporter gene assays for WT or H194R HA-HIF-2α. Plasmid doses are indicated at the bottom. In controls, total DNA dose was normalized with pcDNA3. Luciferase activity was normalized to a Renilla luciferase internal transfection control. *n* = 6 (*H*) or 3 (*I*) per group; *P < 0.05, ***P < 0.001. Anti-HA or anti-Flag western blots for the constructs comparing expression are shown in insets. In (*D*) and (*G*)–(*I*), means ± SEM are shown. Data analyzed by unpaired two-tailed *t*-test in (*D*) and (*G*), and by one-way ANOVA and Tukey's post hoc test in (*H*) and (*I*).

The Andean SNV rs570553380 (H194R) affects a residue that resides in the highly conserved PAS domain of HIF-2α. HIF-2α and ARNT both contain basic helix–loop–helix (bHLH) and PAS domains (fig. 2*A*). The PAS domain, in turn, contains two PAS folds—PAS-A and PAS-B. Both the bHLH and PAS domains are involved in heterodimer formation and DNA binding (Wu et al. 2015). His-194, the site of the Andean HIF-2α mutation encoded by rs570553380, is a highly conserved amino acid located in the region between the PAS-A and PAS-B folds. The 3D structure of the bHLH and PAS domains of HIF-2α bound to those of ARNT reveals that the two proteins wrap around each other asymmetrically (Wu et al. 2015). His-194 is close to the interface between the two proteins (fig. 2*B*).

To examine whether the H194R mutation might affect the interaction between HIF-2α and ARNT, we prepared in vitro translated wild-type (WT) or H194R HIF-2α, mixed each with immobilized in vitro translated ARNT and pulled down ARNT. As shown in figure 2*C* (top panel, compare lanes 2 and 4) and figure 2*D*, we found that the H194R mutation weakens the interaction between HIF-2α and ARNT. In an independent experiment, we transiently transfected HEK293FT cells with constructs for WT or H194R HIF-2α (1–356) and then performed pull-down experiments with glutathione S-transferase (GST) fused to a fragment of ARNT (amino acids 1–485) (fig. 2*E*) and immobilized on glutathione agarose. The HIF-2α and ARNT fragments indicated above both contain the bHLH and PAS domains involved in heterodimer formation (fig. 2*A*). In accord with the previous experiment, WT HIF-2α (1–356) binds to GST-ARNT (1–485), and this interaction is diminished by the H194R mutation (fig. 2*F* and *G*). These data provide evidence that the Andean mutation weakens the interaction between HIF-2α and ARNT.

To examine the transcriptional activity of HIF-2α, we cotransfected HEK293FT cells with expression constructs for full-length WT or H194R HIF-2α along with a luciferase reporter gene driven by three copies of a hypoxia response element (HRE) derived from the human *EPO* gene (Yu et al. 2001). The HRE binds to the HIF-2α:ARNT heterodimer. The HIF-2α constructs were prepared in the context of a P531A mutation that abolishes the primary site of prolyl hydroxylation and thereby stabilizes the HIF-2α under normoxia (Percy et al. 2008). This allows for the assessment of HIF-2α activity without the necessity of hypoxia exposure, which would concurrently activate endogenous HIF-1α and HIF-2α and thereby confound interpretation of results. We find that the H194R mutation results in weakened HIF-2α activity as reflected by luciferase activity (fig. 2*H*). To rule out the possibility that the stabilizing P531A mutation might in some way alter the behavior of the Andean H194R mutation, we also performed reporter gene assays with cells transfected with full-length WT or H194R HIF-2α (lacking the P531A mutation) under normoxic conditions. Similar to the results above, H194R HIF-2α displays weaker transcriptional activity than WT HIF-2α (fig. 2*I*). Together with the protein:protein interaction assays, these reporter gene assays support Andean *HIF2A* being a hypomorphic allele.

### Andean *Hif2a* Has Partial Loss of Function Properties in a Mouse Knockin Model

To examine the functional effect of the H194R mutation in vivo, a knockin mouse was generated using CRISPR technology. In brief, a gRNA targeting exon 6 of the murine *Hif2a* (*Epas1*), Cas9 mRNA, and a single-stranded oligonucleotide donor DNA was injected into C57BL/6 fertilized oocytes. A male mouse with homozygous knockin of the H194R mutation was obtained. Sequencing of five potential off-target loci failed to reveal any off-target effects (primer sequences provided in supplementary table S6, Supplementary Material online). This mouse was bred to a female C57BL/6 mouse, and germline transmission was obtained, resulting in *Hif2a*^H194R/+^ heterozygotes. Heterozygotes were then mated to generate *Hif2a*^H194R/H194R^ mice (supplementary fig. S3, Supplementary Material online). These mice were obtained in close to Mendelian ratio (0.23, 165 pups genotyped; expect 0.25). The mouse line was maintained in a C57BL/6 background.

**Fig. 3.**
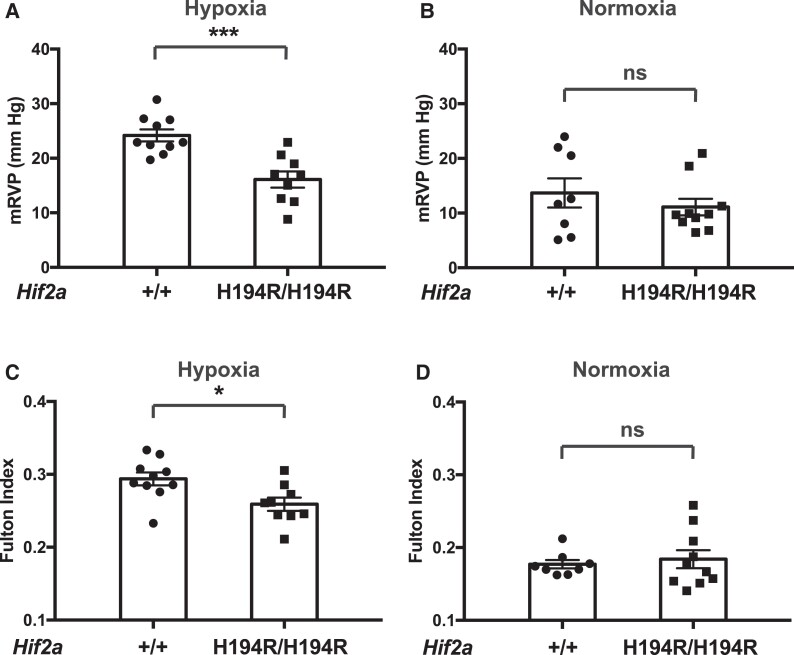
*Hif2a* ^H194*R*/H194*R*^ mice display protection against hypoxia-induced pulmonary hypertension. (*A* and B) mRVP and (*C* and *D*) Fulton index were measured in mice with the indicated *Hif2a* genotypes exposed to either (*A* and *C*) hypoxia (3 weeks of 10% O_2_) or (*B* and *D*) maintained under normoxia. Mice were 3–4 months of age. *P < 0.05, ***P < 0.001, ns, not significant. Data analyzed by unpaired two-tailed *t*-test. Numbers of mice in each group were as follows: (*A* and *C*) +/+ = 10 (five males and five females) and H194R/H194R = 9 (four males and five females); (*B* and *D*) +/+ = 8 (four males and four females) and H194R/H194R = 10 (five males and five females).

We exposed *Hif2a*^H194R/H194R^ or control *Hif2a*^+/+^ mice to 3 weeks of hypoxia (10% O_2_) in a custom-made plexiglass chamber with ports (Song et al. 2020). Following this 3-week exposure, which is a well-established procedure for inducing pulmonary hypertension in wild-type C57BL/6 mice, we measured mean right ventricular pressure (mRVP) of the mice by right heart catheterization and measured the Fulton index (weight of right ventricle [RV]/weight of left ventricle [LV] + septum [S]; a measure of right ventricular hypertrophy). As shown in figure 3*A* and *C*, both mRVP and the Fulton index are significantly decreased in *Hif2a*^H194R/H194R^ mice as compared with *Hif2a*^+/+^ mice. As controls, neither mRVP nor the Fulton index shows differences between *Hif2a*^+/+^ and *Hif2a*^H194R/H194R^ mice maintained under normoxia (fig. 3*B* and *D*).

**Fig. 4.**
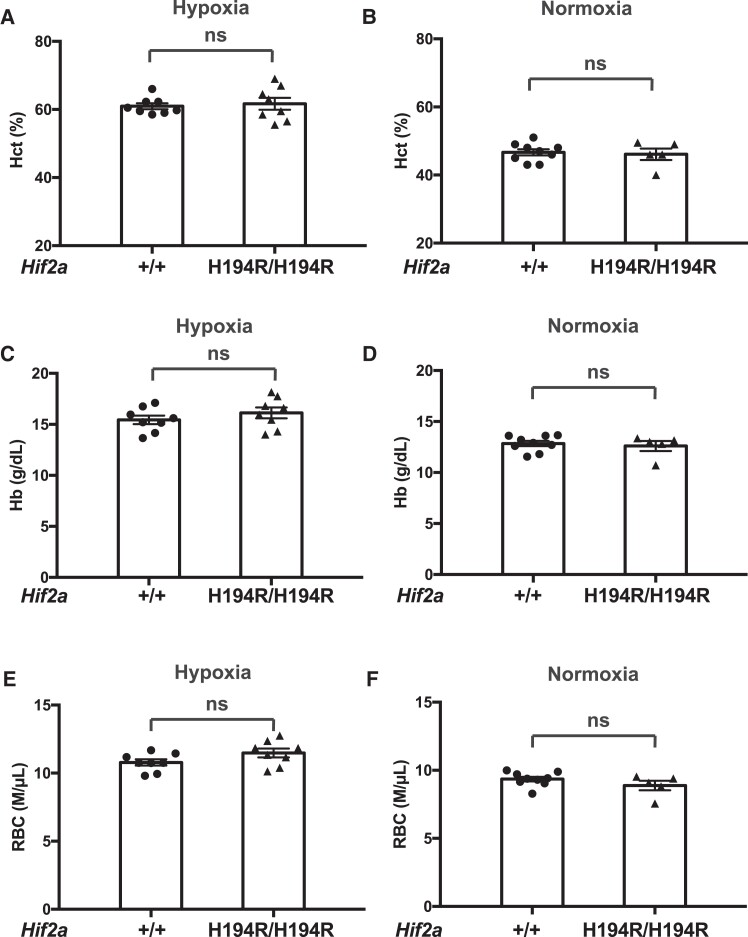
*Hif2a* ^H194*R*/H194*R*^ mice do not display changes in red cell indices. (*A* and *B*) Hct, (*C* and *D*) Hb concentration, and (*E* and *F*) red cell count were measured in mice with the indicated *Hif2a* genotypes exposed to either (*A*, *C*, and *E*) hypoxia (3 weeks of 10% O_2_) or (*B*, *D*, and *F*) maintained under normoxia. Mice were 2–3 months of age. ns, not significant. Data analyzed by two-tailed *t*-test. Numbers of mice in each group were as follows: (*A*, *C*, and *E*) +/+ = 8 (three males and five females) and H194R/H194R = 8 (three males and five females); (*B*, *D*, and *F*) +/+ = 9 (four males and five females) and H194R/H194R = 5 (two males and three females).

The hematocrit (Hct), Hb concentration, and red blood cell counts (RBC) in mice after hypoxia treatment or under normoxia were no different between these two groups (fig. 4), which demonstrates a dissociation between the mRVP and Hct responses to the Hif-2α mutation, at least in this experimental context. Indeed, previous studies employing *Hif2a* haploinsufficient mice have shown that these mice are also protected against hypoxia-induced pulmonary hypertension, but not hypoxia-induced erythrocytosis (Brusselmans et al. 2003; Hodson et al. 2016). We did not observe any differences in white blood cell count (WBC) or platelet count (Plt) between *Hif2a*^+/+^ and *Hif2a*^H194R/H194R^ mice after hypoxia treatment or under normoxia (supplementary fig. S4, Supplementary Material online). Taken together, our data provide evidence that the *Hif2a*^H194R^ mutation protects mice against hypoxia-induced pulmonary hypertension and right ventricular hypertrophy.

**Fig. 5.**
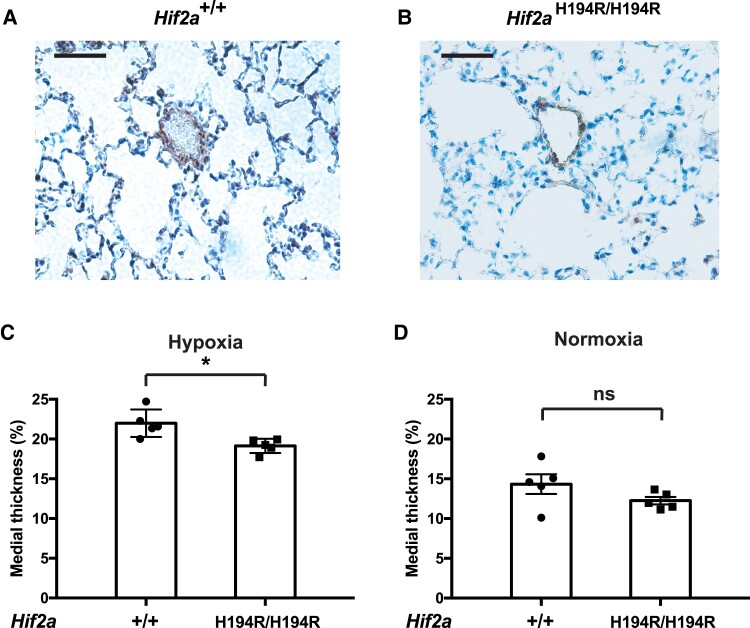
*Hif2a* ^H194*R*/H194*R*^ mice display decreased small pulmonary artery thickness following exposure to hypoxia. (*A* and *B*) Photomicrographs of immunoperoxidase-stained sections of lung using antibodies against smooth muscle actin from wild-type and *Hif2a*^H194*R*/H194*R*^ mice exposed to hypoxia (3 weeks of 10% O_2_). Small pulmonary arteries are in the center of each photomicrograph. Bars indicate 50 µm. (*C* and *D*) The medial thickness of small pulmonary arteries (expressed as a percentage of the external diameter) was measured in lungs obtained from mice with the indicated *Hif2a* genotypes exposed to either (*C*) hypoxia (3 weeks of 10% O_2_) or (*D*) maintained under normoxia. Mice were 3 months of age. *P < 0.05; ns, not significant. Data analyzed by two-tailed *t*-test. Numbers of mice in each group were as follows: (*C*) +/+ = 5 (two males and three females) and H194R/H194R = 5 (two males and three females); (*D*) +/+ = 5 (two males and three females) and H194R/H194R = 5 (two males and three females). Each data point represents a single animal.

We obtained lung tissue from *Hif2a*^+/+^ and *Hif2a*^H194R/H194R^ mice after 3 weeks of hypoxia exposure (10% O_2_) and stained sections with antibodies against smooth muscle α-actin to visualize small arteries (fig. 5*A* and *B*). Medial thickness of small pulmonary arteries was lower in *Hif2a*^H194R/H194R^ mice than in control mice (fig. 5*C*). We did not observe differences in this measurement between these mice when maintained under normoxia (fig. 5*D*).

**Fig. 6.**
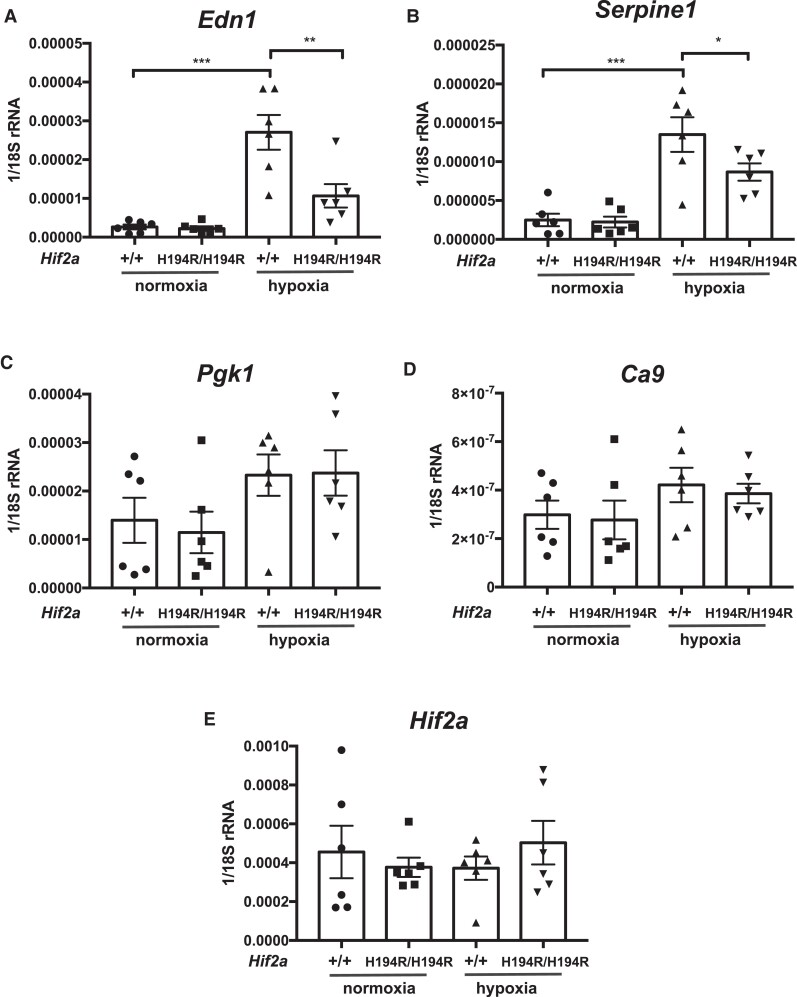
(*A*–*E*) *Hif2a*^H194*R*/H194*R*^ mice display decreased levels of hypoxia-induced *Edn1* mRNA transcripts in the lung. The levels of the indicated mRNAs (relative to 18*S* rRNA) in the lung were determined by real-time PCR. Mice were 2–3 months of age and were exposed to either normoxia or hypoxia (48 h of 8% O_2_). *P < 0.05, **P < 0.01, ***P < 0.001. Data analyzed by one-way ANOVA with Tukey's post hoc test. Numbers of mice in each group were as follows: +/+ normoxia = 6 (four males and two females); H194R/H194R normoxia = 6 (four males and two females); +/+ hypoxia = 6 (four males and two females); H194R/H194R hypoxia = 6 (four males and two females).

We isolated RNA from the lungs of *Hif2a*^+/+^ and *Hif2a*^H194R/H194R^ mice exposed to either normoxia or hypoxia (8% O_2_ for 2 days), prepared cDNA, and then performed real-time PCR analyses of selected HIF target genes. Hypoxia induces pulmonary expression of the Hif-2α target *Edn1*, as expected, and we observed that levels of *Edn1* mRNA were lower in *Hif2a*^H194R/H194R^ as compared with wild-type mice (fig. 6*A*). A similar result was seen with the Hif-2α target *Serpine1* (fig. 6*B*). We did not observe any differences between wild-type and *Hif2a*^H194R/H194R^ mice with Hif-1α targets such as *Pgk1* and *Ca9* (fig. 6*C* and *D*). No differences were seen in *Hif2a* transcript level under either normoxia or hypoxia (fig. 6*E*)

## Discussion

Andeans have resided on the Andean Altiplano, which stretches some 600 miles and has an average altitude of about 4,000 m, for roughly 11,000 years (Rademaker et al. 2014). At this altitude, the oxygen partial pressure is less than two-thirds of that at sea level. Yet Andeans have flourished under these harsh environmental conditions, displaying physiological and genetic evidence for natural selection. However, the functional adaptive changes responsible for the Andean pattern of hypoxia adaptation remain elusive, leaving a critical gap in our understanding of the genetic changes that contribute to Andean altitude-adaptive phenotypes. Here, we provide evidence that the Andean HIF-2α missense point mutation, H194R, is a hypomorphic allele, which supports the hypothesis that high-altitude hypoxia has led to Andean–Tibetan convergent evolution in the form of hypomorphic *HIF2A* alleles.

The present study provides evidence that an Andean *HIF2A* allele is a hypomorphic allele due to impaired heterodimerization with ARNT, resulting in decreased HIF-2α transcriptional activity (fig. 2). Studies of the mouse line with the *Hif2a*^H194R/H194R^ genotype provide independent evidence that it is a hypomorphic allele as this line displays decreased hypoxia-induced pulmonary mRNA levels of *Edn1*, a Hif-2α target gene (fig. 6*A*). Importantly, this mouse also displays protection against hypoxia-induced pulmonary hypertension (fig. 3), and this may be one reason that this allele could be under early-stage selection in Peruvian Andeans. HIF-2α has recently emerged as a promising target for the treatment of pulmonary hypertension (Pullamsetti et al. 2020). The protection from hypoxia-induced pulmonary hypertension observed in our mouse model also provides evidence that the Andean *HIF2A* allele is a hypomorphic allele. *Hif2a* haploinsufficiency and endothelial cell conditional knockout of *Hif2a* are protective (Brusselmans et al. 2003; Kapitsinou et al. 2016; Tang et al. 2018), whereas a hypermorphic *Hif2a* knockin mutation that stabilizes the protein leads to pulmonary hypertension under normoxia (Tan et al. 2013). Our genotype–phenotype association results showing a significant correlation between H194R genotype and FeNO support the hypothesis that the H194R allele is a hypermorphic allele that leads to increased levels of the vasodilator NO. AG heterozygotes displayed higher mean FeNO values than AA homozygotes, and GG homozygotes showed higher mean FeNO values than AG heterozygotes. However, it should be noted that the average log-transformed FeNO value for individuals with the GG genotype was 2.49 ppb. This mean FeNO was based on seven GG individuals, two of whom had high values of FeNO (3.64 and 3.53 ppb) and five of whom had values ranging from 1.79 to 2.48 ppb, which were lower than the average FeNO for AG heterozygotes (mean log FeNO = 2.49 ppb). Future studies will be needed to confirm whether this allele is associated with increased FeNO among Andeans. It will be particularly important to explore this association in a larger cohort with additional GG homozygous individuals. Future studies also will be needed to confirm whether the Andean *HIF2A* allele is associated with PAP in high-altitude Andeans. In this regard, we have not observed any changes in transcripts of *Arginase1* or *Arginase2*, genes that influence NO production, in the *Hif2a*^H194R/H194R^ mice compared with controls (data not shown). We also note that our studies do not rule out the possibility that other *HIF2A* SNVs, including ones described above (supplementary table S1, Supplementary Material online), might contribute to Andean high-altitude adaptation.

Interestingly, the *Hif2a*^H194R/H194R^ mice do not display any difference in Hct, Hb concentration, or red cell count compared with wild-type mice following hypoxia exposure (fig. 4). This may suggest a differential effect of the *Hif2a*^H194R^ allele on the pulmonary vasculature as opposed to the Epo-producing cells of the kidney. Alternatively, this may relate to the experimental conditions employed or to intrinsic differences between mice and humans in their response to HIF-2α loss of function. It is relevant to note that some groups have reported no difference between wild-type and Hif-2α haploinsufficient mice in terms of hypoxia-induced erythrocytosis (Brusselmans et al. 2003; Hodson et al. 2016), whereas other groups have reported that Hif-2α haploinsufficient mice display blunted hypoxia-induced erythrocytosis (Scortegagna et al. 2005; Peng et al. 2017). Whether this disparity is due to differences in the length of hypoxia exposure, the mouse strain background, or some other variable is not known. Another consideration is that these studies, as well as our studies, were performed under normobaric hypoxia, which is not identical to hypobaric hypoxia (Coppel et al. 2015). That being said, it should be noted that a study in humans did not find any difference between the erythrocytosis induced by normobaric versus hypobaric hypoxia (Hauser et al. 2016).

We did not detect an association between the Andean H194R allele and Hb cocentration in this cohort of Peruvian Andeans. This finding is in contrast to studies conducted among Tibetans where noncoding *HIF2A* variation has been associated with lower Hb concentration (Beall et al. 2010; Yi et al. 2010; Peng et al. 2017; Yang et al. 2017). There are at least two possible explanations for this discrepancy. First, association studies conducted among a larger cohort of Peruvian Andeans or Andeans from a wider geographic and ethnic (e.g., Aymara) range may increase power to detect an association between H194R genotype and Hb. Our lack of statistical support for an association does not rule out the possibility that the *H194R* allele may contribute to Andean Hb. Second, it also is possible that there are differences in the behavior between the Andean and Tibetan *HIF2A* alleles that differentially impact their phenotypic consequences. The Andean allele produces a change in the protein sequence whereas the Tibetan allele likely produces a change in transcription. It is possible that the transcriptional effects of the Tibetan allele might have a disproportionately large impact on erythropoiesis induced by the EPO-producing cells of the kidney, while the protein change of the Andean allele may not have a similar effect. The effects of the 30 or so nucleotide substitutions in the Tibetan *HIF2A* allele on *HIF2A* gene transcription in these specialized EPO-producing cells are unknown, so this remains a distinct possibility.

Interestingly, the following lines of evidence suggest that Tibetan *HIF2A* allele also may be a hypomorphic allele. 1) The Tibetan *HIF2A* allele is strongly correlated with low Hb concentration (Beall et al. 2010; Yi et al. 2010; Peng et al. 2017; Yang et al. 2017). 2) *HIF2A* mRNA levels from peripheral blood mononuclear cells, umbilical endothelial cells, and placentas from Tibetans are lower than those from control Han Chinese (Petousi et al. 2014; Peng et al. 2017). As there are no CDS mutations in Tibetan HIF-2α, it is possible that this allele may result in decreases in *HIF2A* transcription or splicing. 3) Tibetans who are homozygous for the Tibetan *HIF2A* allele have a blunted plasma EPO response to hypoxia in comparison to Tibetans who are homozygous for the lowland allele (Petousi et al. 2014). 4) The Tibetan *HIF2A* haplotype is correlated with lowered systolic pulmonary arterial pressure (Peng et al. 2017). 5) In cell culture-based reporter gene assays, select Tibetan *HIF2A* SNVs lead to lower transcriptional activity (Gray et al. 2022). Combined with our data reported here, the observations provide evidence for selection of hypomorphic *HIF2A* alleles in two human high-altitude populations, Tibetans and Andeans.

Notably, in comparison to Tibetans, Andeans are not conventionally thought of as being protected against pulmonary hypertension. One possible explanation is that the age of the Andean H194R allele is younger than the Tibetan *HIF2A* allele. Archeological evidence supports permanent human occupation of the Andean Altiplano for some 11,000 years (Rademaker et al. 2014). While this is certainly ample time for natural selection to have operated on adaptive genomic variation, it is conceivable that the H194R allele is a new mutation that only arose relatively recently in the Andean lineage. This is supported by the observation that the adaptive Peruvian Andean *HIF2A* allele is present at a frequency of 0.09, with a frequency of 0.07 among 1KG PEL, 0.32 in Andean Collas from Argentina, and absent among all other Indigenous American lowland populations considered to date with the exception of Indigenous Argentine lowlanders where the frequency is 0.06 (Eichstaedt et al. 2017). Notably, this is substantially lower than the frequency of about 0.72 for the adaptive Tibetan *HIF2A* allele (with a control population frequency of less than 0.02) (Huerta-Sánchez et al. 2014; Peng et al. 2017). As discussed above, it is also conceivable that the functional strength of the Andean and Tibetan *HIF2A* alleles may differ, possibly in a cell type–specific manner. Finally, it is worth emphasizing that HIF-2α influences many physiologic responses beyond pulmonary vascular tension and erythrocytosis, including ventilation, iron metabolism, and angiogenesis, as well as pathologic processes such as tumorigenesis. Thus, the overall fitness effect of these alleles that affect a transcription factor with pleiotropic effects will likely depend on the balance between beneficial and (potentially) deleterious effects.

*HIF2A* is emerging as a common gene under selection in multiple high-altitude species, which include North American high-altitude deer mice, Andean horses and ducks, and Tibetan dogs, horses, goats, pigs, chickens, and loaches (Wang et al. 2015; Li et al. 2017; Graham and McCracken 2019; Liu et al. 2019; Ma et al. 2019; Witt and Huerta-Sánchez 2019). Much remains to be learned. In the majority of cases, it is not known whether the *HIF2A* allele is a hypermorphic or hypomorphic allele. It is also unclear what phenotype is being selected. Critically, there is a lack of knowledge of the mechanisms underlying these alleles. A notable exception is high-altitude deer mouse Hif-2α that is characterized by a T755M mutation (Schweizer et al. 2019). This mutation is a hypomorphic mutation that impairs binding of Hif-2α to its transcriptional coactivator Creb-binding protein, which is a histone acetyltransferase that promotes the formation of active chromatin (Song et al. 2021). This partial loss of function is consistent with the reduced ventilatory sensitivity following chronic hypoxia in these mice (Ivy et al. 2022) because it previously has been observed that inducible genetic loss of Hif-2α activity strongly attenuates the increased hypoxic ventilatory response (HVR) induced by chronic hypoxia, and conditional deletion of Hif-2α in the carotid body severely impairs HVR (Hodson et al. 2016; Macias et al. 2018). It remains to be seen how the T755M mutation relates to the increased heart rate under hypoxia that has been observed in these animals (Schweizer et al. 2019). It is also not known whether this *Hif2a* allele is associated with differences in blood pressure in the pulmonary vasculature.

From this study and previous work, we conclude that downregulation of HIF-2α activity in high-altitude species can occur by at least two distinct mechanisms—inhibition of heterodimerization with ARNT in the case of Andeans and inhibition of the transcriptional activation domain in the case of the high-altitude deer mice. It is likely that Tibetan downregulation of HIF-2α activity might occur by a third mechanism—transcriptional downregulation, although it must be emphasized that the mechanism has not been precisely characterized. Alterations in the *HIF2A* gene in high-altitude species therefore may represent convergent evolution occurring by distinct molecular mechanisms. An interesting comparison is with a different example of convergent evolution, lactase persistence, in which the adaptive SNVs cluster in a region upstream of the *LCT* gene, in the gene *MCM6* (Tishkoff et al. 2007; McIntosh and Scheinfeldt 2012; Liebert et al. 2017), and may therefore act by a common mechanism, namely transcriptional regulation.

## Materials and Methods

### Participant Recruitment

Andean participants self-identified as Quechua-born and raised at high altitude. They were recruited in Cerro de Pasco, Peru (4,338 m, population ∼80,000), through radio advertisements and word of mouth among the community. Male and female study participants were unrelated, healthy, nonpregnant/lactating, nonsmokers, and between the ages of 18 and 35 who were screened for anemia using altitude-specific cutoff values. None of the study participants were mine workers even though Cerro de Pasco is a mining town. Study participants provided whole blood for DNA extraction and phenotypic measurements including Hb concentration and FeNO. Hb concentration was assessed using a HemoCue (Angelholm, Sweden) blood Hb analyzer. FeNO was measured using a NIOX Mino (Aerocrine, Uppsala, Sweden) according to the manufacturer's instructions. Indigenous Mexican study participants born and raised at low altitude spoke Tzeltal, Tzotzil, or Ch’ol and were recruited in and around the city of Palenque, Chiapas, Mexico (13 m, population ∼43,000). All study participants provided written informed consent at the time of enrollment. The study was approved by the institutional review boards at the University of California Los Angeles (IRB # 20-001141), University of Michigan (IRB # HUM00064387), Syracuse University (IRB # 10-138), the Universidad Peruana Cayetano Heredia, and the Centro de Investigación y Docencia Económicas.

### H194R Genotyping and WGS

H194R (rs570553380, c.581A>G) was genotyped using PCR coupled with diagnostic restriction enzyme digestion (TspRI enzyme). Heterozygotes for the AG genotype were confirmed with Sanger sequencing at the University of Michigan's DNA Sequencing Core.

WGS data were generated at the University of Michigan's DNA Sequencing Core using an Illumina NovaSeq and a single S4 flow cell. Samples were sequenced using 75 base paired-end reads at 15× depth of coverage. Samples were demultiplexed and trimmed using Trim Galore! v.0.6.7. Low-quality reads with a default Phred score of 20 were removed (Schmieder and Edwards 2011). Reads were aligned to the human reference sequence, hg38, using the Burrows–Wheeler Alignment in BWA (Li and Durbin 2010). Variant calls were performed in GATK (DePristo et al. 2011). For further quality control, reads were filtered using a mapping quality >30 with VCFtools, and variant sites or individuals missing more than 10% of data were removed using Plink2.0 (Danecek et al. 2011; Chang et al. 2015).

### Selection Tests

We applied three methods to identify signatures of natural selection: 1) PBS (Yi et al. 2010); 2) the two-population haplotype-based statistic (XP-nSL) (Szpiech et al. 2021); and 3) a composite test of selection that combines PBS with the haplotype test nSL to detect beneficial alleles at intermediate frequencies (15–50%) (Ferrer-Admetlla et al. 2014; Eichstaedt et al. 2017). PBS compared Peruvian Andeans (*n* = 46) to 1KG Han Chinese from Beijing (CHB, *n* = 112) and Mexican Maya (*n* = 48). Pairwise *F*_ST_ was calculated using Weir–Cockerham's equation (Weir and Cockerham 1984), with a genome-wide empirical distribution generated using 6,639,921 SNVs and a significance threshold of *α* = 0.01. Data were phased using SHAPEIT (Delaneau et al. 2008). WGS data were lifted over using CrossMap v0.6.4 to GRCh37 (hg19) for haplotype test computation, including nSL and XP-nSL (Szpiech and Hernandez 2014; Zhao et al. 2014). XP-nSL results were normalized using genome-wide normalization in Selscan (Voight et al. 2006; Szpiech and Hernandez 2014), and an empirical significance threshold of *α* = 0.01 was used.

To estimate the composite test of selection (Eichstaedt et al. 2017), we first calculated PBS and nSL using standard procedures, with nSL scores calculated for all SNPs with a MAF > 0.05 then normalized using allele frequency bins in Selscan (Szpiech and Hernandez 2014). Next, we calculated genome-wide significance based upon the absolute value of normalized nSL scores standardized in derived allele frequency (DAF) bins. nSL scores were sorted by intermediate DAF (15–50%) and PBS values were filtered for those that matched with these intermediate nSL scores. Both of these PBS and nSL values were then transformed into empirical *P* values based upon 4% DAF bins. We merged the *P* values of these two dependent statistical tests using the harmonic mean *P* value (HMP) (Wilson 2019). This method for merging the *P* values differs from the original Eichstaedt et al. (2017) method wherein Fisher's method was used to merge *P* values. We chose to use the HMP instead of Fisher's method as it works to combine separate tests performed on dependent data sets whereas Fisher's method is robust for combining separate tests completed on independent data sets. The HMP is only reliable when individual *P* values are <0.5, and so any PBS or nSL scores with *P* values greater than 0.5 were removed. We filtered our 15% ≤ DAF ≤ 50% data set to consider only the top 5% of extreme value, as the appropriate threshold for a well-calibrated HMP is 0.05 (Wilson 2019). CADD and DANN scores were used to predict the functional impact of each SNV.

### Association Testing

Genome-wide SNP data were generated using the Affymetrix (Santa Clara, CA) Axiom Biobank Genotyping Array for all Peruvian Andean and Mexican Maya study participants. We performed a PCA using 405,419 variants that were filtered for linkage disequilibrium (*r*^2^ > 0.8) and genotyping rate (<0.1) in Plink 2.0 (Purcell et al. 2007; Chang et al. 2015) (fig. 1). PCA included publicly available data from the 1000 Genomes Project for 60 Yorubans (YRI), 45 CHB, 45 Japanese from Tokyo (JPT), and 60 individuals of north-central European ancestry (CEU) from the Human Genome Diversity Project-Centre d’Etude du Polymorphisme Humain (HGDP-CEPH) (1000 Genomes Project Consortium). Participant relatedness was estimated using KING (Manichaikul et al. 2010) in PLINK 2.0. Three individuals related at the first-degree level and six individuals related at the second-degree level were identified and excluded from genotype–phenotype association testing. Standard linear regression was performed in PLINK using dominant and additive models of inheritance (Purcell et al. 2007). Regression coefficients were calculated for the minor allele. Participant sex and the first five PCs were included as covariates in the models.

### In Vitro Translation

In vitro transcribed and translated proteins were prepared using the TnT T7 Quick Coupled Transcription/Translation System (Promega). Reactions were conducted at 30 °C for 90 min. The following templates were used: pcDNA3-HA-HIF-2α, pcDNA3-HA-HIF-2α H194R, and pcDNA3.1-Flag-ARNT. Plasmid construction is described in supplementary Material, Supplementary Material online.

### Immunoprecipitations

In vitro translated Flag-ARNT (2 µl) was incubated with 30 µl aliquots of anti-Flag (mAb M2) agarose (Sigma) in a total volume of 500 µl of buffer A (50 mm Tris, pH 8, 150 mm NaCl, and 1% Triton X-100) supplemented with 0.1% bovine serum albumin and rocked for 1 h at 4 °C. The resins were washed 2× with buffer A and then incubated with in vitro translated HA-HIF-2α or HA-HIF-2α H194R (10 µl) in a total volume of 500 µl of buffer A and rocked for 1 h at 4 °C. The resins were washed 3× with buffer A and eluted, and the eluates were subjected to SDS–PAGE and western blotting.

### Cell Culture

HEK293FT cells were maintained in DMEM/10% FBS/100 IU/ml penicillin/100 µg/ml streptomycin and transfected using Lipofectamine 2000 as described (Song et al. 2013). HEK293FT cells were obtained from Invitrogen.

### GST Pull-Down Assays

GST and GST-ARNT (1–485) were purified from *Escherichia coli* DH5α transformed with pGEX-5X-1 and pGEX-ARNT (1–485), respectively, using affinity chromatography on glutathione (GSH)-Sepharose (Cytiva). Transfected HEK293 cells were lysed in buffer A supplemented with mammalian protease inhibitor cocktail (Sigma P8340). The lysates were clarified by centrifugation at 15,800 × g for 10 min at 4 °C. GST or GST-ARNT (1–485) prebound to 30 µl of GSH-Sepharose was incubated with cellular lysates for 1 h at 4 °C. The resins were washed 3× with buffer A and eluted, and the eluates were subjected to SDS–PAGE and western blotting.

### Western Blotting

The sources of antibodies to Flag tag and HA tag have been described (Arsenault et al. 2013; Song et al. 2013). The mouse mAb to GST (P1A12) was developed by E.A. Wayner and M. Linial and was obtained from the Developmental Studies Hybridoma Bank developed under the auspices of the NICHD, National Institutes of Health and maintained by the University of Iowa Department of Biology (Iowa City, IA). The procedure for western blotting has been described (Song et al. 2013). CDP-Star (Millipore Sigma) was employed as a substrate, and quantitation was performed using ImageJ software (National Institutes of Health).

### Luciferase Reporter Gene Assays

HEK293FT cells in 96-well plates were transfected with plasmids using Lipofectamine 2000. Luciferase assays were performed using a Dual-Glo Luciferase Assay System and a Glomax Explorer microplate reader (Promega). Firefly luciferase activity was normalized to Renilla luciferase activity expressed from a pRL-TK internal transfection control.

### Mouse Lines

*Hif2a* ^H194R^ mice were generated using CRISPR technology. A guide RNA plasmid, pUC57-sgRNA-Hif2a, was constructed by subcloning into the BsaI site of pUC57-sgRNA (a gift from Dr. Xingxu Huang, Addgene plasmid # 51132), a duplex consisting of the following two oligonucleotides: 5ʹ TAGGCCCCAGGTCCTGCACTGCAC 3ʹ and 5ʹ AAACGTGCAGTGCAGGACCTGGGG 3ʹ. A single-stranded oligodeoxynucleotide donor (Ultramer grade) was synthesized by IDT and consisted of the following sequence: 5ʹ GTCTCAAGAAAGAGCCAGGAGCAGGAGGTGCCTGAGGCCTCTCCCTCTTCTCGGCCGTCTCGGCCTTGTCTTACTTCTGTGCTTTGACCCCAGGTCTTAAGATGCACCGGGCAAGTGAGAGTCTACAACAACTGCCCCCCTCACAGTAGCCTCTGTGGCTCCAAGGAGCCCCTGCTGTCCTGCCTTATCATCATGTGTGA 3ʹ. A construct to express Cas9, pBS-SK-Cas9, has been described (Song et al. 2020). *Hif2a* gRNA was transcribed from DraI-linearized pUC57-sgRNA-Hif2a using a MEGAshortscript T7 kit (Ambion). *Cas9* polyadenylated mRNA was transcribed from XbaI-linearized pBS-SK-Cas9 using a mMESSAGE mMACHINE T7 Ultra Transcription kit (Ambion). Both *Hif2a* gRNA and *Cas9* mRNA were purified using a MEGAclear kit (Ambion). A mixture of *Hif2a* gRNA (50 ng/µl), *Cas9* mRNA (100 ng/µl), and donor oligonucleotide (100 ng/µl) was injected into the cytoplasm of fertilized C57BL/6J oocytes by the University of Pennsylvania Transgenic & Chimeric Mouse Facility. From this procedure, a male mouse with homozygous knockin of the *Hif2a*^H194R^ allele was obtained. The mouse was bred to a C57BL/6J female, and a mouse with a heterozygous knockin was obtained. Sequencing of five potential off-target loci, one with two mismatches to the gRNA sequence and four with three mismatches (there were no off-target loci with zero or one mismatch), did not reveal any off-target effects of the gRNA. The sequences of the primers to examine potential off-target effects are provided in supplementary table S6, Supplementary Material online. This mouse was bred to C57BL/6J female mice to obtain additional heterozygotes. Heterozygote crosses yielded *Hif2a*^H194R/H194R^, *Hif2a*^H194R/+^, and *Hif2a*^+/+^ mice at frequencies of 23.0%, 48.5%, and 28.5%, respectively, close to Mendelian ratios (165 pups genotyped). These mice were maintained in a C57BL/6J background. All animal procedures were approved by the Institutional Animal Care and Use Committees at the University of Pennsylvania in compliance with Animal Welfare Assurance. Approximately equal numbers of males and females were employed for the experiments.

### PCR Genotyping

DNA was isolated from mouse tails. For genotyping, a common 5ʹ primer and knockin- or WT-specific 3ʹ primers were used. The following primers were employed: mHif2 HDR 5ʹ: 5ʹ GGCACAGCAACAAAGGGACACC 3ʹ; mHif2 HDR 3ʹ: 5ʹ CACTTGCCCGGTGCATCTTAA 3ʹ (for *Hif2a*^H194R^); and mHif2 HDR 3ʹ WT: 5ʹ CACTTGCCCGGTGCAGTGCAG 3ʹ (for *Hif2a*^+^). The PCR product for both knockin and WT alleles = 0.40 kb.

### Hypoxia Experiments

Mice were placed in a custom-made 330-l (100 × 60 × 55 cm) plexiglass chamber with ports. Hypoxia was achieved by infusing nitrogen into the chamber with the use of a ProOx 360 Controller to achieve the desired oxygen concentration, with fan circulation and continuous oxygen monitoring. Carbon dioxide was monitored continuously with a TIM10 CO_2_ monitor (CO_2_Meter) and maintained below 0.3% by port vents and inclusion of a beaker containing soda lime (a CO_2_ scavenger) within the chamber. Mice were monitored daily, and mouse cages changed weekly.

### Right Ventricular Pressure Measurements

For right ventricular pressure measurements, mice were anesthetized by isoflurane inhalation, placed in the supine position, and a midline cervical incision was made to expose the right jugular vein by microsurgical techniques. A 1.4-French miniaturized pressure catheter (Millar instruments) was inserted via the right jugular vein and advanced to the RV chamber for the measurement of RV pressure and heart rate. Data were recorded and analyzed using PowerLab equipment (ADInstruments) and LabChart 8 software. The mRVP measurements were made by the Penn Cardiovascular Institute Rodent Cardiovascular Phenotyping Core, which was blinded to the identity of the mice.

### Fulton Index Measurements

Atria were dissected from ventricles, and then the RV was separated from the LV and S. The Fulton Index = RV/(LV + S).

### Hematology Analysis

Blood samples were obtained from the inferior vena cava and were collected in Microvette 100 LH heparin-coated collection tubes (Sarstedt). Hct was measured manually using a StatSpin MP Centrifuge (Beckman Coulter). Hematology analysis was conducted using a Hemavet 950FS Hematology Analyzer (Erba Diagnosistics).

### Histologic Analysis

Fixation of mouse lungs, preparation of slides for antismooth muscle α-actin immunohistochemistry, and assessment of medial thickness of pulmonary arteries were performed as described (Tan et al. 2013).

### Real-Time PCR

RNA was isolated from lung tissue using TRIzol, and cDNA synthesis and real-time PCR were performed as described (Li et al. 2010; Arsenault et al. 2013).

### Statistical Analysis of In Vitro Experiments and Mouse Studies

Data were analyzed by either unpaired Student's *t*-test or by one-way ANOVA with Tukey's post hoc test (GraphPad Prism 7). Differences were considered significant when *P* < 0.05. Unless otherwise specified, data are presented as means ± SEM.

Details on plasmids are available in Supplemental Methods, Supplementary Material Online.

## Supplementary Material

msad162_Supplementary_Data

## Data Availability

Whole genome sequencing data for HIF2A are available through Dryad (https://datadryad.org/, DOI:10.5068/D1VH68). Other data underlying this manuscript are available in the manuscript and in its Supplementary Material online.
