# Supplementary material for: High-Altitude Andean H194R HIF2A Allele Is a Hypomorphic Allele
Source: Mol Biol Evol. 2023 Jul 18;40(7):msad162. doi: 10.1093/molbev/msad162 (PMC10370452; doi:10.1093/molbev/msad162)
Supplement: msad162_Supplementary_Data [file msad162_supplementary_data.zip › Hif2Asupp.docx]

**Supplemental Material**

**High-Altitude Andean H194R *HIF2A* Allele is a Hypomorphic Allele**

Kelsey Jorgensen^1*^, Daisheng Song^2*^, Julien Weinstein^3^, Obed A. Garcia^4^, Laurel N. Pearson^5^, Maria Inclan^6^, Maria Rivera-Chira^7^, Fabiola Leon-Velarde^7^, Melisa Kiyamu^7^, Tom Brutsaert^8,^ Abigail W. Bigham^1β^, Frank S. Lee^2β^

^1^Department of Anthropology, University of California, Los Angeles, California, USA

^2^ Department of Pathology and Laboratory Medicine, University of Pennsylvania Perelman School of Medicine, Philadelphia, PA, USA.

^3^Department of Anthropology, The University of Michigan, Ann Arbor, Michigan, USA

^4^Department of Biomedical Data Science, Stanford University, Stanford, California, USA

^5^Department of Anthropology, The Pennsylvania State University, State College, Pennsylvania, USA

^6^División de. Estudios Políticos, Centro de Investigación y Docencia Económicas

^7^Departamento de Ciencias Biológicas y Fisiológicas, Universidad Peruana Cayetano Heredia, Lima, Peru

^8^Department of Exercise Science, Syracuse University, Syracuse, New York, USA

* = These authors contributed equally to this work

β = Corresponding authors

**Supplemental Results**

Out of 32 significant SNVs in *HIF2A* and 50KB upstream and downstream of the CDS coordinates, the top four SNVs for PBS were the intergenic variants rs148755828 (PBS = 0.14, P_E_ = 0.0004), rs4953338 (PBS = 0.13, P_E_ = 0.0009), rs73926244 (PBS = 0.13, P_E_ = 0.0009), and rs114479604 (PBS = 0.12, P_E_ = 0.0009) that were less than 50kb upstream from *HIF2A*. Five of these significant SNVs were within *HIF2A*, although the mutation of interest H194R was not included as it was not possible to calculate pairwise F_ST_ on Mexican Maya and Han Chinese with allele frequencies of 0%.

Peruvian Andean individuals were compared to Mexican Maya for XP-nSL. The four significant SNVs identified with XP-nSL were intronic variants rs7565341 (XP-nSL = 2.61, P_E_ = 0.0086), rs7594912 (XP-nSL = 2.57, P_E_ = 0.0092), rs7568285 (XP-nSL = 2.54, P_E_ = 0.0097), and a novel intronic variant without an rsID (hg38 position 46372091, hg19 position 46599230) with (XP-nSL = 2.61, P_E_ = 0.00086).

The composite method combines PBS with nSL calculated for a single population, in this case Peruvian Andeans. This method is powered to detect intermediate frequency sweeps by focusing on missense SNVs with derived allele frequencies (DAF) >~15%, but <50%. Using this method, we identified 14 significant SNVs upstream, downstream, and within the CDS (Supplemental Table S4), all of which were significant using PBS (p<0.01), but fell specifically within an intermediate frequency range of 15% ≤ DAF ≤ 50% in Peruvian Andeans. CADD scores were predicted to be neutral for the 14 significant SNVs, but an algorithm that uses a deep learning approach to annotate pathogenicity, DANN (Quang, et al. 2015), predicted that three of these SNVs (all which are upstream of the *HIF2A* gene) may be deleterious: rs17034920 (DANN = 0.79), rs140448261 (DANN = 0.84), and rs4952816 (DANN = 0.75). The Peruvian Andean frequency of the H194R (rs570553380) missense SNV was 5.6% (0% among Mexican Maya speakers). Therefore, it did not pass the frequency threshold to be included in our composite test of selection.

There were 32 significant SNVs with PBS and four significant SNVs with XP-nSL in *HIF2A* and 50KB upstream and downstream of the CDS coordinates at the 1% level. The missense mutation H194R (rs570553380) was not in LD with any of the SNVs under selection, and not in LD with any alleles in *HIF2A* or 50KB upstream and downstream of the CDS coordinates. For the alleles under selection, three out of the four significant SNVs with XP-nSL were in high LD (R^2^>0.8) with each other: rs7565341, rs7594912, and rs7568285 (Supplemental Table S7) and all four resided on a single haplotype (Supplemental Figure S2D). The 32 significant SNVs with PBS were on multiple haplotypes (Supplemental Figure S2D), and most significant SNVs were clustered in two distinct regions, one upstream and one downstream of *HIF2A* (Figure S2C). There were several SNVs within each of these two clusters that are in high LD (r^2^>0.8) with other significant SNVs located closely in their respective region (Supplemental Table S7).

Of these 36 significant SNVs with PBS and XP-nSL, 24 had known rsIDs and 23 were classified as non-coding intronic or intergenic. Only rs73926246 (PBS = 0.1008, p = 0.0028) was classified as a regulatory region variant predicted to be a potential *HIF2A* enhancer ([www.ensembl.org](http://www.ensembl.org)), with no known documented effects on phenotype in ClinVar, PolyPhen, or available literature. None of these significant SNVs were unique to Peruvian Andeans and all were found in other 1KG global populations at low or intermediate frequency.

**Supplemental Materials and Methods**

**Plasmids**

pcDNA5/FRT/TO-3xFlag-HIF-2α H194R/P531A was constructed in several steps. First, we constructed pBS-SK-HIF-2α H194R EcoR I by overlapping PCR as follows. In the first round of PCR, we used pcDNA5/FRT/TO-3xFlag-HIF-2α P531A (Furlow, et al. 2009) as a template and amplified a 0.7 kb product using the following primers: 5ʹ GCGTGTACGGTGGGAGGTC 3ʹ (CMV 5ʹ) and 5’ actttcacctggcccgtgcaTcTTaagaccttccaggtggctga 3’. Using the same template, we also amplified a 0.34 kb product using the following primers: 5’ tcagccacctggaaggtcttAAgAtgcacgggccaggtgaaagt 3’ and 5’ ccttggtgcacaagttctggt 3’ (HIF2 900 3’). The two PCR products were mixed and employed as a template in a second round of PCR using the CMV 5’ and HIF2 900 3’ primers. The 0.84 kb product was digested with BamH I/EcoR I and subcloned into the BamH I/EcoR I site of pBS-SK to generate pBS-SK-HIF-2α H194R EcoR I. This plasmid was then digested with BamH I/EcoR I, and the 0.8 kb product was subcloned into the BamH I/EcoR I (partially digested) site of pcDNA5/FRT/TO-3xFlag-HIF-2α P531A to generate pcDNA5/FRT/TO-3xFlag-HIF-2α H194R/P531A.

pcDNA3-HA-HIF-2α P531A was constructed by subcloning the 2.6 kb BamH I/Xba I fragment of pcDNA5/FRT/TO-3xFlag-HIF-2α P531A into the BamH I/Xba I site of pcDNA3-HA. pcDNA3-HA-HIF-2α H194R/P531A was constructed by subcloning the 2.6 kb BamH I/Xba I fragment of pcDNA5/FRT/TO-3xFlag-HIF-2α H194R/P531A into the BamH I/Xba I site of pcDNA3-HA.

pcDNA3-HA-HIF-2α was constructed by subcloning the 0.9 kb Hind III/EcoR I fragment of pcDNA3-HA-HIF-2α P531A into the Hind III/EcoR I site of pcDNA3-3xFlag-HIF-2α (Arsenault, et al. 2016). pcDNA3-HA-HIF-2α H194R was constructed by subcloning the 0.9 kb Hind III/EcoR I fragment of pcDNA3-HA-HIF-2α H194R/P531A into the Hind III/EcoR I site of pcDNA3-3xFlag-HIF-2α.

pcDNA5/FRT/TO-3xFlag-HIF-2α (1-356) was constructed by subcloning the 1.1 kb Hind III/Nco I (blunt) fragment of pcDNA5/FRT/TO-3xFlag-HIF-2α (Percy, et al. 2008) into the Hind III/BstX I (blunt) site of pcDNA5/FRT/TO. pcDNA3-HA-HIF-2α (1-356) was constructed by subcloning the 1.1 kb BamH I/Xba I fragment from pcDNA5/FRT/TO-3xFlag-HIF-2α (1-356) into the BamH I/Xba I site of pcDNA3-HA.

pcDNA3-HA-HIF-2α (1-356) H194R was constructed as follows. pcDNA3-3xFlag-HIF-2α H194R constructed by subcloning the 0.8 kb BamH I/EcoR I fragment from pcDNA3-HA-HIF-2α H194R/P531A into the BamH I/EcoR I site of pcDNA3-3xFlag-HIF-2α. pcDNA3-3xFlag-HIF-2α (1-356) was constructed by subcloning the 1.1 kb BamH I/Xba I fragment of pcDNA5/FRT/TO-3xFlag-HIF-2α (1-356) into the BamH I/Xba I site of pcDNA3-3xFlag. pcDNA3-3xFlag-HIF-2α (1-356) H194R was constructed by was constructed by subcloning the 0.8 kb BamH I/EcoR I fragment from pcDNA3-3xFlag-HIF-2α H194R into the BamH I/EcoR I site of pcDNA3-3xFlag-HIF-2α (1-356). pcDNA3-HA-HIF-2α (1-356) H194R was constructed by subcloning the 0.8 kb BamH I/EcoR I fragment of pcDNA3-3xFlag-HIF-2α (1-356) H194R into the BamH I/EcoR I site of pcDNA3-HA-HIF-2α (1-356).

pcDNA3.1-Flag-ARNT (pcDNA3.1-Flag-HIF1B) was a gift from Dr. James Brugarolas (Addgene plasmid #99916). pcDNA3.1-HA-ARNT was constructed by first amplifying by PCR a 0.9 kb DNA product from pcDNA3.1-Flag-ARNT using the following two primers: 5’-GTACAAGCTTACCATGTACCCGTACGACGTGCCGGACTACGCTGGGATCCAGATGGCGGCGACTACTGCCAACCCCGAA-3’ and 5’-agtggaccaccacgaagtgaggtt-3’. The product was digested with Hind III/Blp I and then subcloned into the Hind III/Blp I site of pcDNA3.1-Flag-hARNT.

pcDNA3.1-HA-ARNT (1-485) was constructed by digesting pcDNA3.1-HA-hARNT with PpuM I/Not I, blunting the ends with the Klenow fragment of *E. coli* DNA polymerase, and then religating. pGEX-ARNT (1-485) was constructed by subcloning the 1.5 kb BamH I/Xho I fragment of pcDNA3.1-HA-hARNT (1-485) into the BamH I/Xho I site of pGEX-5X-2.

**Supplemental Figures**

**
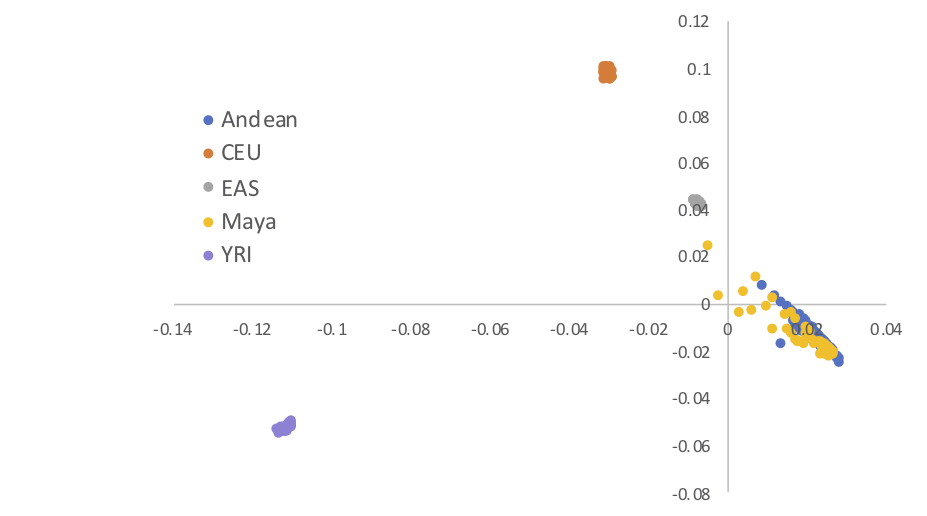
**

**Supplemental Figure S1.** Principal Component Analysis (PCA) of Peruvian Andeans (301), Mexican Maya (100), Centre de Polymorphism Humain (CEPH) Europeans (CEU) (60), East Asian consisting of Han Chinese from Beijing and Japanese from Tokyo (EAS), and Yorubans from Ibadan Nigeria (YRI). The PCA was conducted in Plink1.9 using 354,770 variants that were pruned for linkage disequilibrium (r2>0.8) and with genotyping rates greater than 90%.

**Supplemental Figure S2.** (A) *HIF2A* Exonic Single Nucleotide Variants (SNVs) in Peruvian Andeans and Mexican Maya. The approximate location of each SNV is presented along with its rsID, ancestral/derived alleles, and the Peruvian Andean Minor Allele Frequency (MAF). Synonymous SNVs are shown above the gene schematic whereas missense SNVs are shown below. Total SNVs within the CDS and the 5ʹUTR and 3ʹUTR are presented in the table*.* (B) Population Branch Statistic (PBS) values for Peruvian Andeans within *HIF2A* and 50kb upstream and downstream. Peruvian branch lengths were calculated using Mexican Maya and 1KG Han Chinese as outgroups. Dotted lines indicate the 1% significance level (PBS = 0.072) based on a genome-wide empirical distribution generated from 6,639,921 SNVs. Chromosome location (HG38) is depicted along the x-axis and PBS is on the y-axis. Genes are indicated including their intron and exon structure for each test statistic. (C) Distribution of *HIF2A* XP-nSL values for Peruvian Andeans compared to Mexican Maya speakers. The dotted line indicates the 1% significance level (XP-nSL = 2.529) for positive selection based on a genome-wide empirical distribution for Peruvian Andeans. Chromosome location (HG19) is depicted along the x-axis and the test statistic is shown on the y-axis. (D) Haplotype surrounding HIF2A. Haplotype count is shown in the y-axis. SNV rsID is provided on the x-axis. H194R SNP rs570553380 is shown in red font below the panel and indicated by the red arrow at the top of the panel. HIF2A exon/intron structure is depicted above the x-axis.


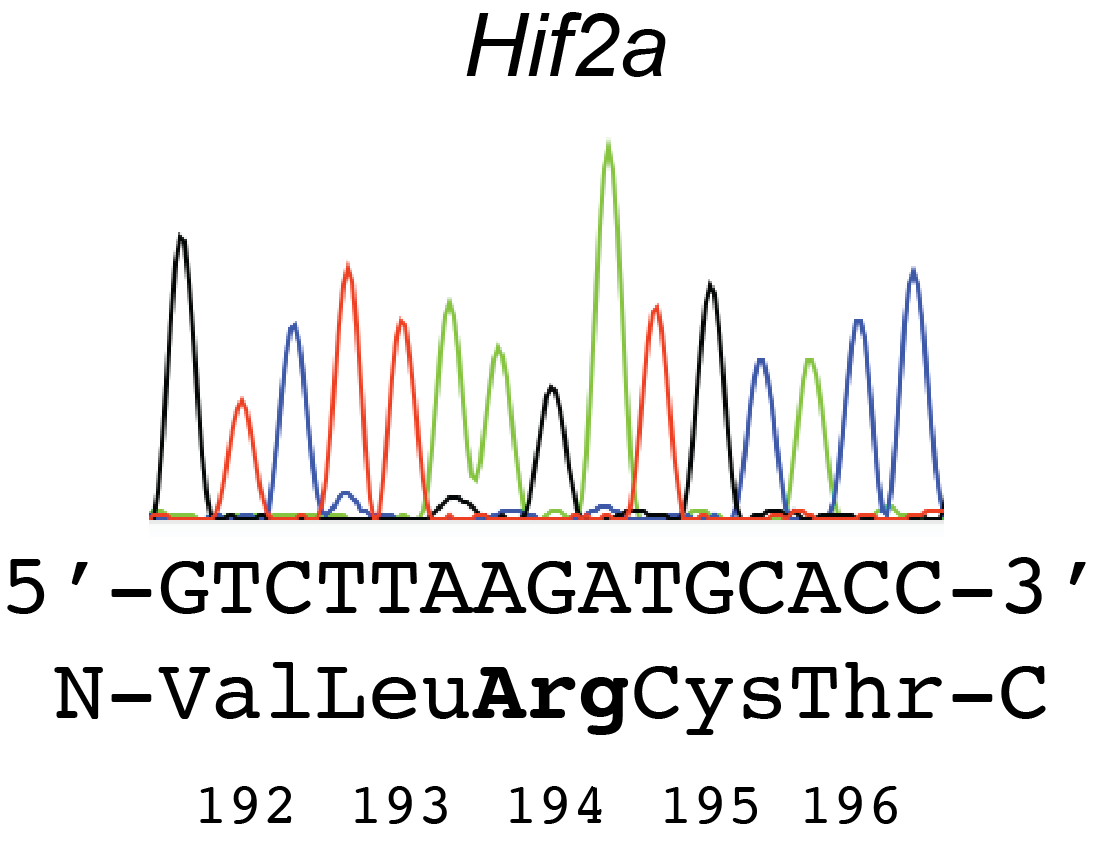


**Supplemental Figure S3. Generation of *Hif2a* H194R knockin mice.** DNA sequencing chromatogram of tail DNA demonstrating homozygous H194R substitution in the *Hif2a* gene. Amino acid numbers are indicated at the bottom.


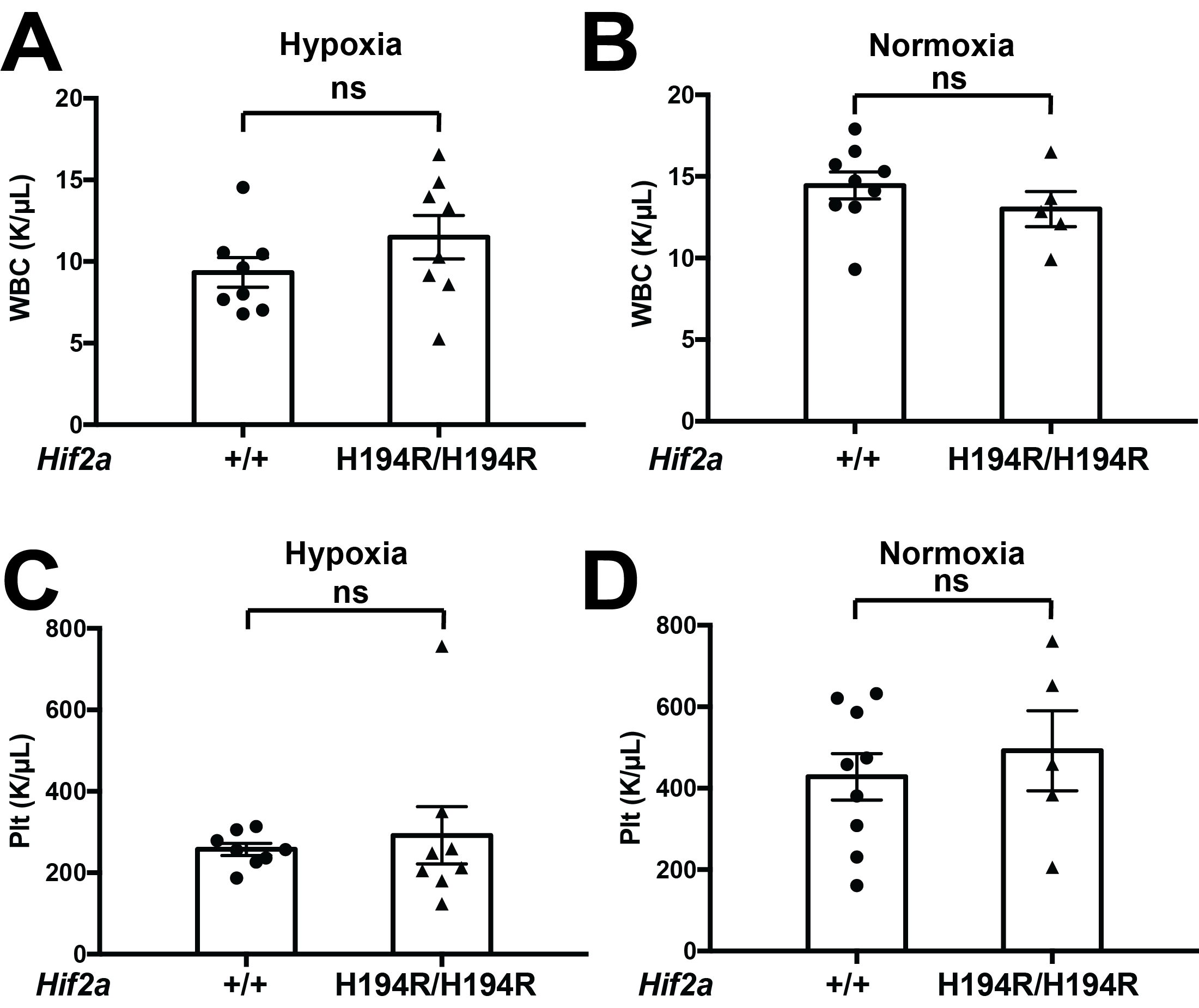


**Supplemental Figure S4. *Hif2a* ^H194R/H194R^ mice do not display changes in white blood cell or platelet counts.** (A and B) White blood cell and platelet (C and D) counts were measured in mice with the indicated *Hif2a* genotypes exposed to either (A, C) hypoxia (three weeks of 10% O_2_) or (B, D) maintained under normoxia. Mice were two to three months of age. ns = not significant. Data analyzed by two-tailed t test. Numbers of mice in each group were as follows: (A and C) +/+ = 8 (3 male, 5 female), H194R/H194R = 8 (3 male, 5 female); (B and D) +/+ = 9 (4 male, 5 female), H194R/H194R = 5 (2 male, 3 female).
